# Dioscin regulates oxidative stress and autophagy in uric acid-induced HK-2 cells through the P62-KEAP1-NRF2 signaling pathway

**DOI:** 10.1186/s12882-025-04311-z

**Published:** 2025-07-29

**Authors:** Jiashu Feng, Weiliang Zhang, Ruiqi Liu, Ting Xiang, Xinlin Wu

**Affiliations:** 1 Department of Traditional Chinese Medicine, The First Affiliated Hospital of Sun Yat-Sen University, Zhongshan 2nd Road, Guangzhou, 510080 P. R. China; 2 Department of Traditional Chinese Medicine, The Second People’s Hospital of Shuangliu District, Sixing Road, Chengdu, 610200 P. R. China

**Keywords:** Uric acid, Dioscin, P62-KEAP1-NRF2 signaling pathway, Oxidative stress, Autophagy

## Abstract

**Objective:**

This study aims to explore the therapeutic effects and mechanisms of dioscin on the UA-induced HK-2 cells fibrosis model by modulating oxidative stress and autophagy.

**Methods:**

HK-2 cells fibrosis was constructed by UA stimulation. CCK8 was used to assess cell proliferation. Flow cytometry was employed to detect apoptosis. MDC staining was performed to observe the formation of autophagosomes. Western blot was used to evaluate the levels of oxidative stress, autophagy and fibrosis markers. The detection of ROS and Elisa assay were used to analyze the changes of oxidative stress.

**Results:**

Dioscin significantly inhibits cell apoptosis. Dioscin increases the expression of LC3, Beclin-1 and NRF2 and decreases the expression of P62 and KEAP1. Furthermore, dioscin inhibits the levels of ROS and MDA, and promotes the levels of SOD and CAT. Moreover, dioscin significantly downregulates the expression of TGF-β, FN, and Collagen I. However, the regulatory effects of dioscin on these indicators are inhibited when NRF2 is knocked down.

**Conclusion:**

These results suggest that dioscin treats the UA-induced HK-2 cells fibrosis model by targeting the modulation of NRF2 to regulate oxidative stress and promote protective autophagy. The mechanism may be associated with the restoration of the P62-KEAP1-NRF2 signaling pathway.

**Trial registration:**

Not applicable.

**Supplementary Information:**

The online version contains supplementary material available at 10.1186/s12882-025-04311-z.

## Introduction

Hyperuricemia (HUA) is a metabolic disorder characterized by an elevated level of uric acid (UA) in the blood. The global prevalence of HUA has witnessed a significant surge in recent years, particularly among the younger demographic [1–4]. HUA can induce renal inflammation, oxidative stress, endothelial and epithelial damage, and cell apoptosis, as well as increase epithelial-mesenchymal transition, leading to various harmful diseases such as uric acid nephropathy (UAN) [5–7]. However, there is currently no specific cure for HUA, and clinical treatments mainly focus on reducing UA levels using drugs such as allopurinol and febuxostat. Unfortunately, these drugs often have poor tolerability and are associated with various adverse reactions [8]. Therefore, there is an urgent imperative to develop novel therapeutics exhibiting enhanced efficacy and reduced toxicological profiles for the treatment of HUA.

The KEAP1-NRF2 signaling pathway is an important endogenous antioxidant pathway in the body. Under normal conditions, NRF2 binds to KEAP1 in the cytoplasm. KEAP1 is the main negative regulator of NRF2 and is involved in the degradation process of NRF2 [9]. However, when exposed to oxidative stress stimuli, conformational changes occur in KEAP1, resulting in dissociation from NRF2 and subsequent activation of transcription for various antioxidant genes. Therefore, the KEAP1-NRF2 signaling pathway can be used as an important target for therapeutic oxidative stress [10].

In addition to oxidative stress, autophagy activity is essential for maintaining the stability, vitality, and physiological function of renal cells [11]. P62 serves as a common biomarker of autophagy and plays a role in lysosomal transport and proteasomal ubiquitination processes, acting as an important upstream regulatory factor in the KEAP1-NRF2 signaling pathway. Excessive levels of UA under oxidative stress have been shown to promote renal fibrosis progression [12]. Previous studies have confirmed that autophagy plays a key role in protecting renal function in experimental models of renal fibrosis (RF) [13]. Activation of the P62-KEAP1-NRF2 signaling pathway has been found to alleviate oxidative stress, enhance autophagy, and provide protection against reperfusion injury in kidney [14]. Therefore, targeting the P62-KEAP1-NRF2 signaling pathway may offer an effective approach for treating HUA.

Dioscin, a naturally occurring steroidal saponin found in various traditional Chinese herbs such as wild yam and Tribulus terrestris, has been identified as the primary bioactive component extracted from these plant [15]. Numerous studies have demonstrated that dioscin possesses pharmacological effects including anti-inflammatory, antioxidant, anti-apoptotic properties, promotion of UA excretion and improvement of UA levels and renal function [16]. It has been demonstrated that dioscin can alleviate renal damage by regulating metabolism [17]. However, no study has yet confirmed the effects of dioscin on RF and HUA, and its underlying mechanisms remain unknown. Therefore, this study aims to investigate whether dioscin can intervene in oxidative stress and autophagy through the regulation of the P62-KEAP1-NRF2 signaling pathway to treat HUA by establishing UA-induced HK-2 cells fibrosis model in vitro and explore its mechanisms.

## Materials and methods

### Cell culture and grouping

The HK-2 cells were purchased from China center for type culture collection (Wuhan, China). Firstly, cells were all cultured in a humidified incubator at 37℃ with DMEM medium containing 10% FBS and 1% penicillin-streptomycin. According to the previous experimental results of the research group, a stable UA-induced HK-2 cells fibrosis model can be prepared by stimulating HK-2 cells with a concentration of 0.5 mg/mL UA for 24 h [18]. For this reason, UA group was induced by UA (0.5 mg/ml, diluted by DMEM) for 24 h.

HK-2 cells were divided into the following 5 groups: Normal control group (NC); UA group and UA + dioscin group, UA + dioscin + siNC group and UA + dioscin + siNRF2 group.

### The time-effect and dose-effect curve of Dioscin

In the UA-induced HK-2 cells fibrosis model, a CCK8 assay was used to plot the time-effect and dose-effect curve of dioscin. Different concentrations of dioscin (50, 100, 200, 400, 800, and 1600 ng/ml) were used for time-effect and dose-effect experiments to determine the optimal intervention concentration and intervention time for the next experiment.

### Cell transfection

siRNA targeting NRF2 (sense primer 5′-GUAGUCCACAUUUCCUUCATT-3′ and antisense primer 5′-UGAAGGAAAUGUGGACUACTT-3′) and control siRNA were purchased from General Biosystems Limited (Anhui, China). Inoculate HK-2 cells in logarithmic growth phase into a 6-well culture plate. When the density of HK-2 cells in the culture plate reaches 70%, 20 nM NRF2 siRNA or control siRNA was transfected with Lipofectamine 8000.

### Cell viability analysis

CCK-8 assay was used to detected cell viability. 1 × 10^4^ cells were seeded and grown in a 96-well plate. Subsequently, cells were treated with dioscin for indicated period (24 h, 48 h and 72 h). The average value of the OD450 was used to determine cell viability.

### Cell apoptosis detection

The Annexin V-FITC/PI assay kit was utilized to assess cellular apoptosis. Following the manufacturer’s protocol, cells were harvested and suspended in binding solution before being incubated with Annexin V-FITC and propidium iodide staining solution for 20 min. Flow cytometry was employed to determine the proportion of apoptotic cells.

### Monodansylcadaverine (MDC) staining assay

Evaluation of autophagosome formation in HK-2 cells through MDC staining. Centrifuge and resuspend HK-2 cells, then stain them with MDC buffer in a cell culture incubator at 37 °C in the dark for 30 min. After washing the HK-2 cells, capture images using a fluorescence microscope and analyze the autophagosomes in HK-2 cells using Image J software.

### Western blot

Total proteins were extracted with RIPA and PMSF (100:1). The protein concentration was measured with a BCA kit. The proteins were separated and performed with 12% and 15% SDS-PAGE gels for electrophoresis, then transferred onto PVDF membranes. After sealing with skimmed milk, add primary antibodies against Beclin-1 (ab210498, Abcam), LC3 (4108 S, Cell Signaling Technology), P62 (ab109012, Abcam), KEAP1 (ab227828, Abcam), NRF2 (ab92946, Abcam), FN (ab268020, Abcam), TGF-β (ab215715, Abcam), Collagen I (ab34710, Abcam) and incubate overnight. Then add secondary antibodies and incubate. Finally, use ECL reagent for luminescent imaging and analyze using ImageJ software.

### Reactive oxygen species (ROS) assay

The ROS levels in HK-2 cells were detected according to the instructions of the ROS assay kit. Before the experiment, HK-2 cells were cultured in a 6 well plate and pretreated with the specified concentration of compounds. After washing with PBS, HK-2 cells were incubated with DCFH-DA fluorescent probe (10 µmol/L) at 37 °C for 30 min. The fluorescence intensity was observed using a fluorescence microscope.

### ELISA

Determination of oxidative stress markers were performed using Elisa kits to measure the activities of malondialdehyde (MDA), catalase (CAT) and superoxide dismutase (SOD) in HK-2 cells, following standard procedures. The concentrations were calculated using a standard curve.

### Statistical analysis

The data presented as Mean ± SD was analyzed using GraphPad Prism 9.2.0 software. Unpaired Student’s t-tests or one-way ANOVA were employed to assess differences between groups. *p* < 0.05 were considered to indicate statistical significance.

## Results

### Dioscin modulated UA-induced cells apoptosis and autophagy

**Fig. 1:**
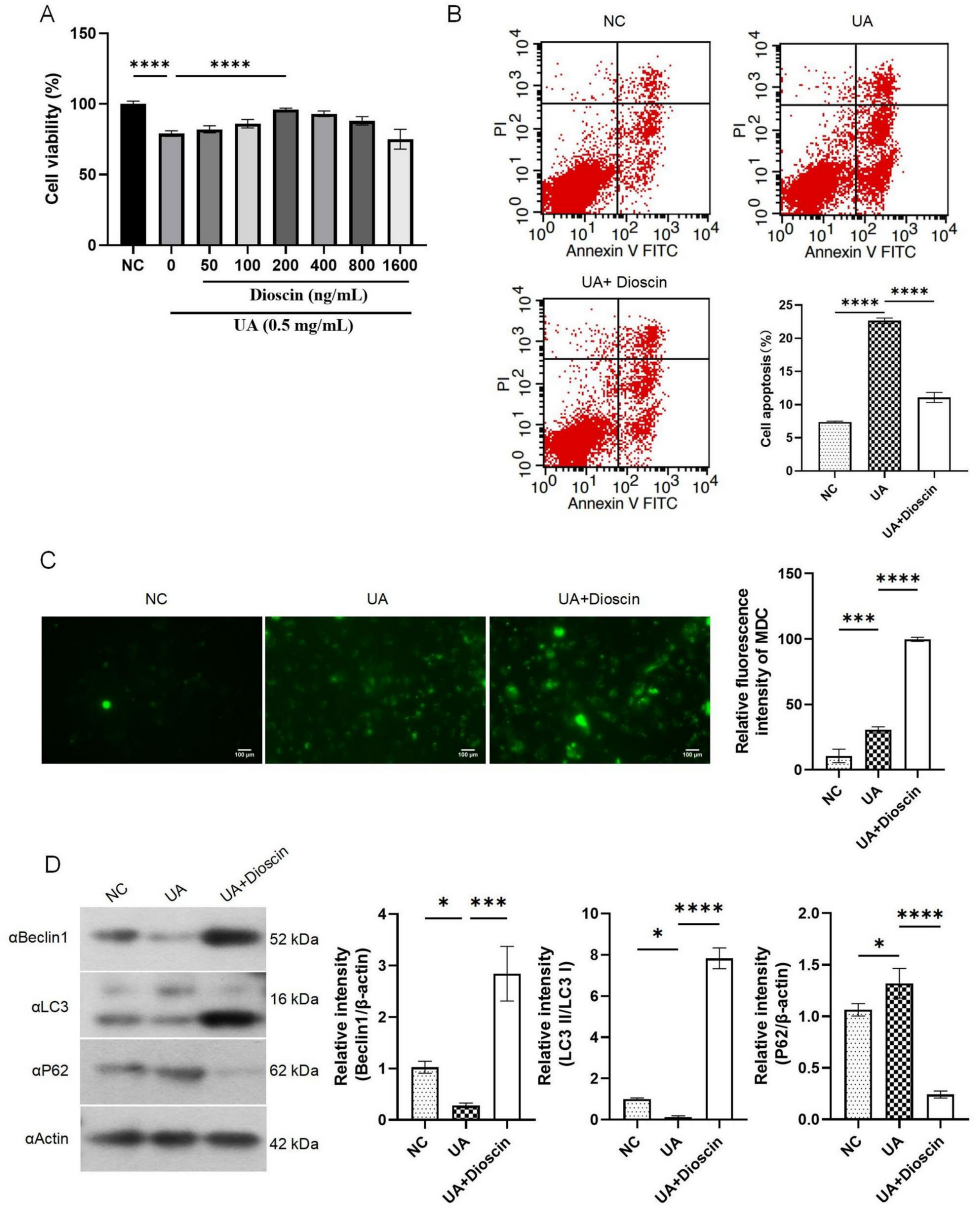
Dioscin regulated UA-induced cell apoptosis and autophagy. Except for the NC group, cells from all other groups were treated with UA for 24 h. Subsequently, the UA + Dioscin group was exposed to dioscin for a duration of 48 h. **A**: Cell proliferation assay; **B**: Cell apoptosis analysis; **C**: MDC staining was employed to observe the autophagosomes; **D**: Detection of autophagy-related proteins. Data are presented as mean ± SD. Statistical significance was obtained by one-way ANOVA. *p < 0.05, ***p < 0.001, ****p < 0.0001

Our preliminary experimental results showed that 0.5 mg/mL of UA significantly inhibited HK-2 cells proliferation, which can establish a stable HK-2 cell fibrosis mode [18]. According to the CCK8 experiments and IC50 detection, under the intervention of dioscin at a concentration of 200 ng/ml for 48 h on UA-induced HK-2 cells, the growth inhibition of HK-2 cells is minimal. (Fig. 1A).

Additionally, we examined UA-induced apoptosis and found that compared with UA group, dioscin treatment significantly reduced the occurrence of cell apoptosis (*P* < 0.0001) (Fig. 1B). MDC can specifically mark autophagosomes. Results from MDC staining indicated that dioscin treatment enhanced cellular autophagy (Fig. 1C). In order to further investigate the molecular mechanism of dioscin in activating autophagy, we use Western blot to detect the expression levels of autophagy-related markers including Beclin-1, P62 and LC3. Results suggested that dioscin promoted Beclin-1 and LC3 expression while inhibiting P62 expression, which means that dioscin may help cells avoid apoptosis through activating protective autophagy (Fig. 1D).

### Dioscin alleviates UA-induced HK-2 cells cellular oxidative stress

**Fig. 2:**
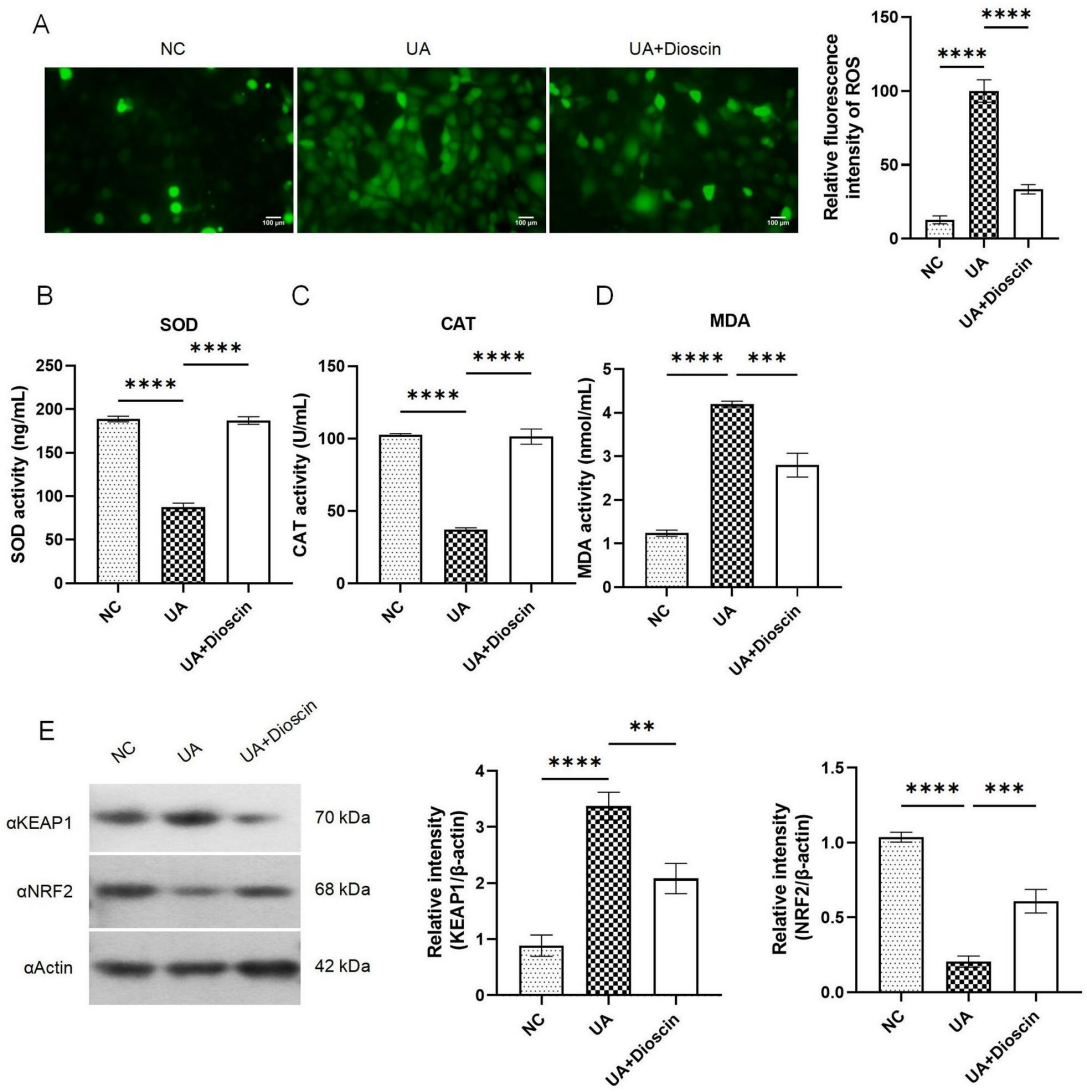
Dioscin alleviated UA-induced cellular oxidative stress. Except for the NC group, cells from all other groups were treated with UA for 24 h. Subsequently, the UA + Dioscin group was exposed to dioscin for a duration of 48 h. **A**: ROS detection; **B**-**D**: Oxidative stress-related biomarkers SOD, CAT, and MDA were quantified using Elisa kits; **E**: Western blot were used to analyze the expression of KEAP1 and NRF2. Data are presented as mean ± SD. Statistical significance was obtained by one-way ANOVA. **p < 0.01, ***p < 0.001, ****p < 0.0001

For purpose of confirming the antioxidant ability of dioscin, we use fluorescence to detect ROS levels, the results demonstrated that UA induces ROS generation, while dioscin effectively attenuates ROS levels (Fig. 2A). Furthermore, results of ELISA revealed dioscin suppressed MDA expression and restored SOD and CAT expression levels significantly (*P* < 0.001 or *P* < 0.0001) (Fig. 2B-D), these indicated the excellent antioxidant stress ability of dioscin. Further analysis of the expression of key molecules KEAP1 and NRF2, which are involved in oxidative stress, revealed that dioscin downregulated the protein expression of KEAP1 and upregulated the protein expression of NRF2 (Fig. 2E). These findings suggested that dioscin could reulate KEAP1-NRF2 to possesses the ability of modulating oxidative stress in UA-induced HK-2 cells fibrosis model.

### Dioscin inhibits fibrosis in UA-induced HK-2 cells fibrosis model

**Fig. 3:**
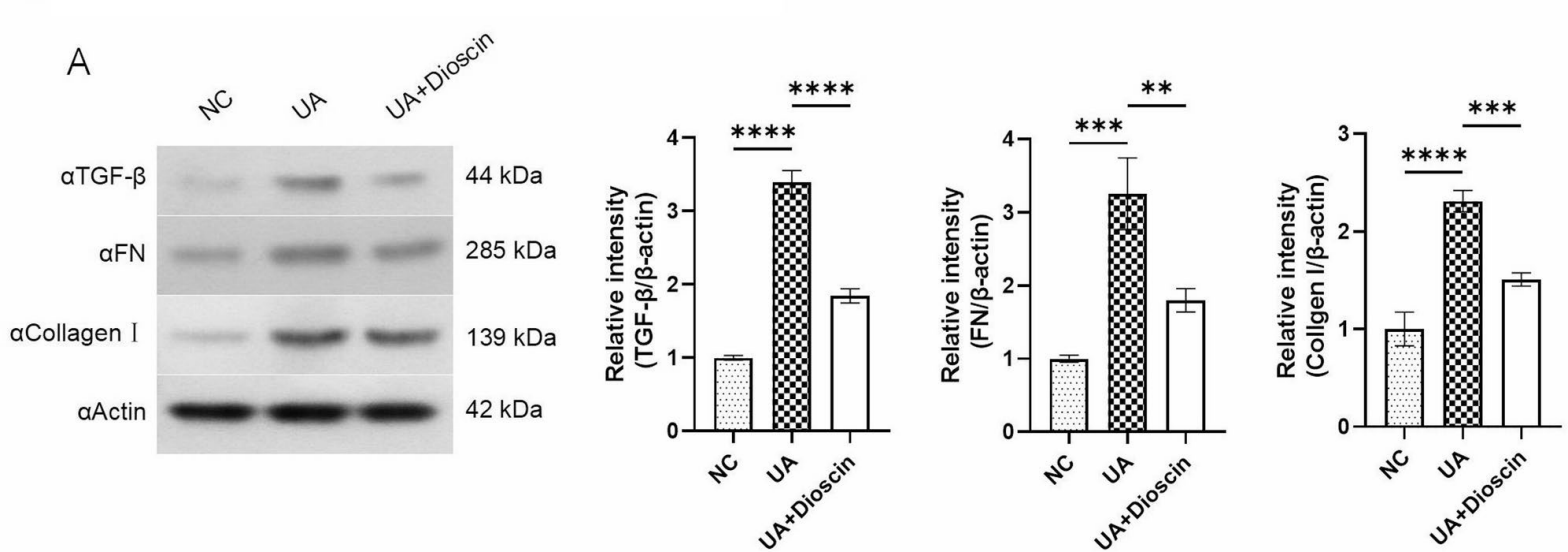
Dioscin reduced UA-induced cell fibrosis. Except for the NC group, cells from all other groups were treated with UA for 24 h. Subsequently, the UA + Dioscin group was exposed to dioscin for a duration of 48 h. **A**: Western blot were used to analyze the expression of TGF-β, FN and Collagen I. Data are presented as mean ± SD. Statistical significance was obtained by one-way ANOVA. **p < 0.01, ***p < 0.001, ****p < 0.0001

To investigate the regulatory ability of dioscin on renal fibrosis, we validated the expression of fibrosis-related factors such as TGF-β, FN and Collagen I through Western blot. According to the results of Western blot, the expression of TGF-β, FN, and Collagen I in the UA group was significantly increased compared to the NC group. However, after treatment with Dionscin, the expression of TGF-β, FN, and Collagen I was significantly decreased compared to the UA group (Fig. 3A). This indicates that the fibrosis degree in the UA group is significantly aggravated compared to the NC group, while after treatment with Dionscin, the fibrosis degree is significantly improved, suggesting that Dionscin has a beneficial effect on improving fibrosis.

### Knockdown of NRF2 counteracted the renoprotective effects of Dioscin in UA induced-HK-2 cells fibrosis model

**Fig. 4:**
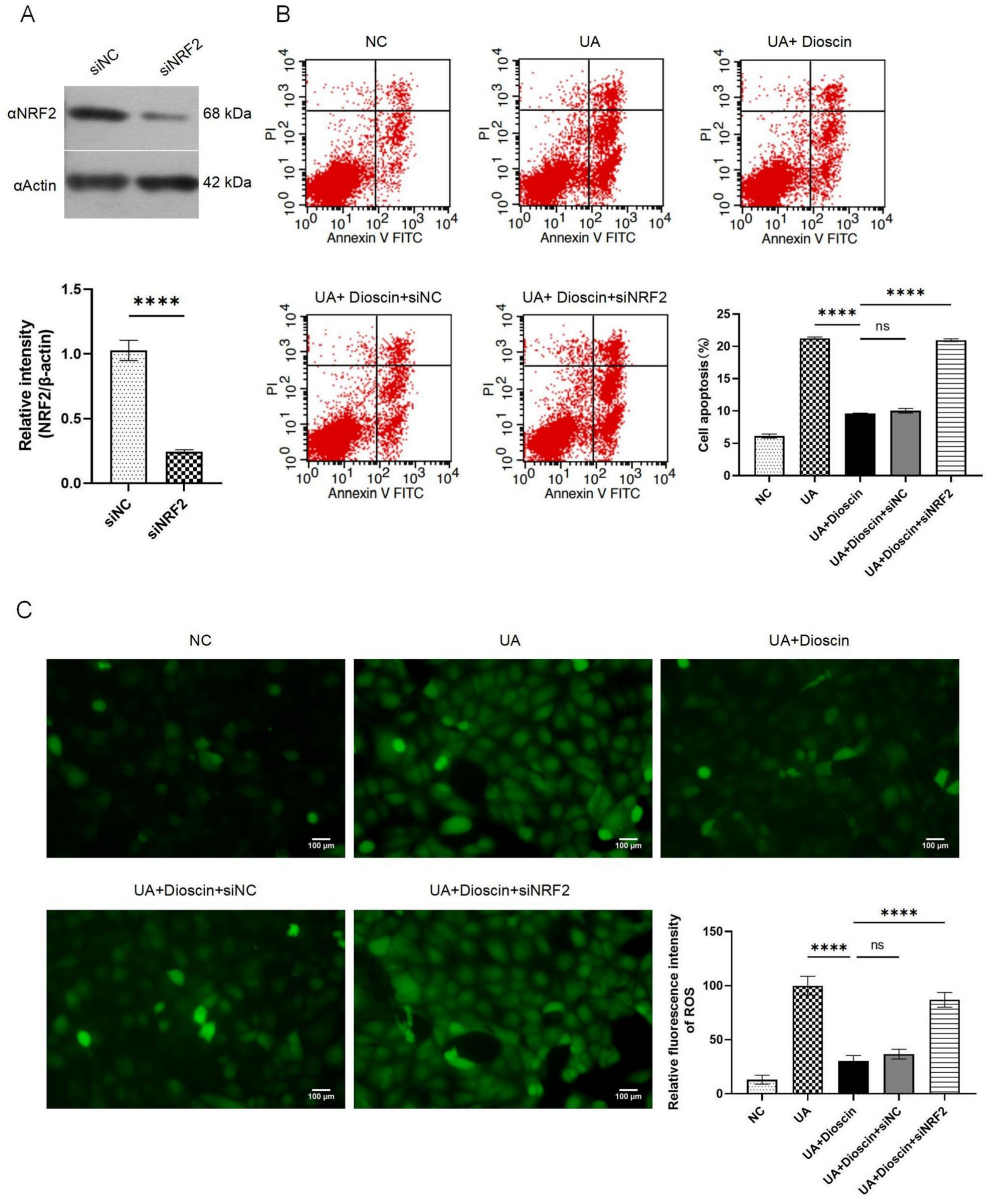
Dioscin targeted KEAP1-NRF2 signaling pathway to mitigate UA-induced cell apoptosis and oxidative stress. Except for the NC group, other cells were transfected with siNRF2 or siNC for 12 h prior to stimulation with UA for 24 h. Subsequently, the UA + Dioscin group was exposed to dioscin for a duration of 48 h. **A**: Efficiencies of NRF2 knockdown in HK-2 cells; **B**: Cell apoptosis analysis; **C**: ROS detection. Data are presented as mean ± SD. Statistical significance was obtained by Student’s t test or one-way ANOVA. ns, no significance, ****p < 0.0001

**Fig. 5:**
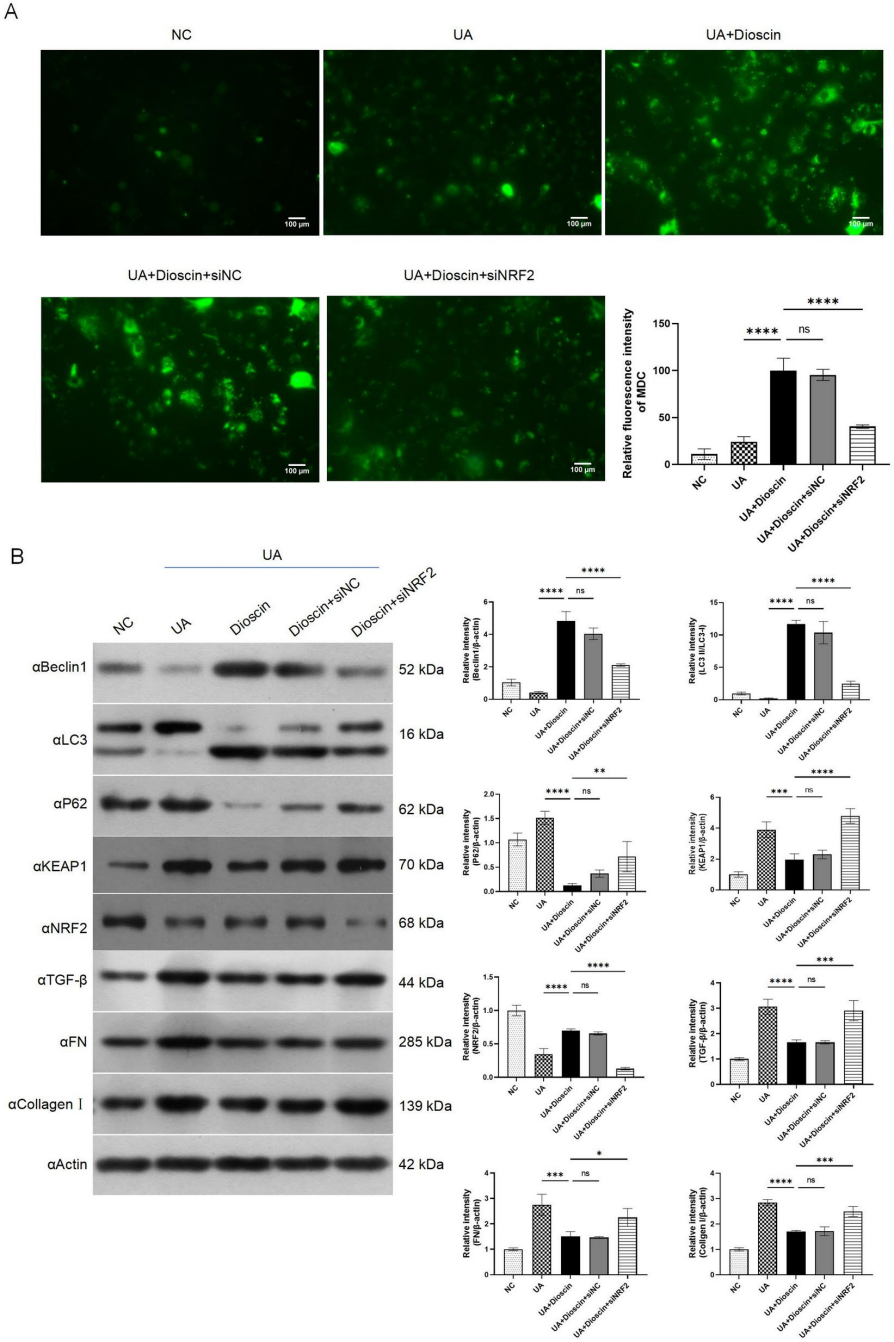
Dioscin targeted KEAP1-NRF2 signaling pathway to attenuate UA-induced autophagy. Except for the NC group, other cells were transfected with siNRF2 or siNC for 12 h prior to stimulation with UA for 24 h. Subsequently, the UA + Dioscin group was exposed to dioscin for a duration of 48 h. **A**: MDC staining was employed to observe the autophagosomes; **B**: Western blot were used to analyze the expression of Beclin1, LC3, P62, KEAP1, NRF2, TGF-β, FN and Collagen I. Data are presented as mean ± SD. Statistical significance was obtained by one-way ANOVA. ns, no significance, *p < 0.05, **p < 0.01, ***p < 0.001, ****p < 0.0001

To further confirm the role of NRF2 in the dioscin-mediated renoprotective mechanism, we established NRF2 siRNA HK-2 cells, the genetic depletion was verified (Fig. 4A). The results showed that NRF2 was required for dioscin to regulate apoptosis (Fig. 4B). Meanwhile, knockdown of NRF2 greatly counteracted the antioxidant stress effect of dioscin (Fig. 4C). Compared with dioscin treatment group, the ability of dioscin in activating protective autophagy was canceled out in NRF2-konckdown HK-2 cells (Fig. 5A). In addition, the role of dioscin in downregulate the factor of autophagy or fibrosis like Beclin-1, LC3, P62, TGF-β, FN and Collagen I were nullified (Fig. 5B).

**Fig. 6:**
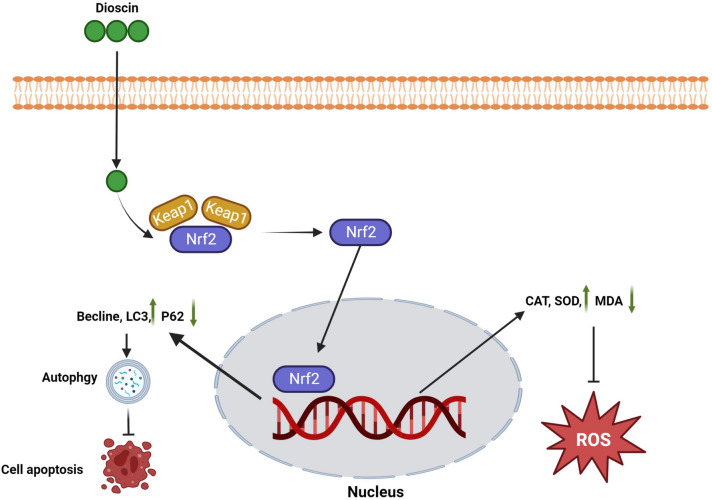
Mechanism of dioscin in UA-induced cell apoptosis and oxidative stress. Dioscin-induced enhancement of NRF2 in HUA cell model promoted autophagy and relieved UA-induced cell apoptosis and oxidative stress

In summary, NRF2 may be a hub of P62-KEAP1-NRF2, it is also the target spot of dioscin in curing UA induced-HK-2 cells fibrosis model, knocking down NRF2 interrupted the protective mechanism of dioscin in UA induced-HK-2 cells fibrosis model, eliminates the ability in activating autophagy, resisting oxidative stress and anti-fibrosis (Fig. 6).

## Discussion

As people’s living standards improve and their diet, environment, and genetic factors change, the prevalence of HUA has been increasing year by year. HUA is not only a sign and pathological result of renal function decline, but also an independent risk factor for the development and progression of renal diseases, seriously endangering human health [19, 20]. Currently, HUA has become one of the major health problems worldwide [21]. Traditional Chinese medicine has always played a pivotal role in the management of chronic diseases, with dioscin being implicated in the regulation of diverse conditions including diabetes [22–24], cardiovascular disorders [25, 26], renal dysfunction [27–29], and arthritis [30, 31]. By modulating NLRP3 inflammasome and NF-κB signaling pathways, dioscin ameliorate kidney damage induced by lupus nephritis [32]. Moreover, dioscin mitigates renal fibrosis through suppression of immune reactions mediated by the NF-κB signaling pathway [28]. Nevertheless, our comprehension regarding the molecular mechanisms underlying dioscin remains limited and high-quality clinical trials assessing their safety and efficacy are yet to be conducted. Our study revealed that dioscin exerts a protective role in the UA-induced HK-2 cell fibrosis model by specifically targeting NRF2 to regulate oxidative stress and autophagy.

The KEAP1-NRF2 signaling pathway is an important signaling pathway that maintains the balance of oxidative stress and redox in the body [33]. NRF2 is the main factor that regulates the balance of oxidative and antioxidative reactions in the body and mediates cellular oxidative stress. Activating NRF2 can reduce oxidative stress and delay the progression of kidney damage [34]. Under normal physiological conditions, KEAP1 and NRF2 form a complex to inhibit NRF2, which exists at a low level in the cytoplasm [33]. When the body is under oxidative stress, the conformation of KEAP1’s cysteine residues changes, causing the bound NRF2 to dissociate and enter the nucleus to bind with ARE, thereby enhancing the cell’s ability to resist oxidative stress and reducing the damage of oxidative stress to cells and tissues [35, 36]. In this study, after treatment with dioscin, compared with UA group, the expression of SOD, CAT, and NRF2 proteins in the UA + dioscin group increased, while the expression of ROS, MDA, and KEAP1 proteins decreased significantly. This indicates that dioscin can alleviate the oxidative stress level lead by UA induced-HK-2 cells fibrosis model. In addition, Oxidative stress plays an important role in renal fibrosis, and its related signaling pathways are closely related to renal fibrosi [37]. Oxidative stress is a major pathogenic factor in chronic kidney disease-related renal fibrosis [38].

Autophagy is crucial in the stress-adaptive response of renal injury. Autophagy is a cellular process that degrades damaged cellular components and regulates cell death and proliferatio [39]. P62 is a ubiquitin-binding autophagy receptor protein that can connect the NRF2 antioxidant signaling pathway and autophagy. It is an important regulatory factor upstream of the KEAP1-NRF2 signaling pathway [40]. P62 can directly interact with KEAP1, mediating the degradation of KEAP1 through the autophagy pathway, causing NRF2 to dissociate from KEAP1 and accumulate stably in the nucleus [41]. Therefore, P62-KEAP1-NRF2 forms a positive feedback loop for antioxidant reactions. The results of the experiment showed that after treatment with dioscin, the expression of P62 protein decreased, while the levels of LC3, Beclin-1, and autophagy increased, and the level of cell apoptosis was inhibited. This indicates that dioscin activates the autophagy level induced by UA induced-HK-2 cells fibrosis model and plays a significant protective role. Studies have shown that activating NRF2 and autophagy can improve the damage of HUA to cells [42]. Other studies have suggested that simultaneous activation of autophagy and NRF2 can play a more effective role in regulating ROS compared to activating autophagy alone [43]. If NRF2 is silenced or knocked out, the cell’s ability to resist oxidative stress is reduced. In our experiment, we found that dioscin can simultaneously activate NRF2 and induce the production of autophagy, thereby alleviating the oxidative stress level induced by UA induced-HK-2 cells fibrosis model by intervening in the P62-KEAP1-NRF2 signaling pathway. However, after knocking down NRF2, the protective effect of dioscin on cell apoptosis was greatly weakened, and its regulatory effect on oxidative stress and autophagy-related factors and proteins was also disrupted, indicating that the antioxidant stress and activation of autophagy by dioscin in UA induced-HK-2 cells fibrosis model are regulated by NRF2. Furthermore, Autophagy plays a role in promoting renal cell survival and combating renal fibrosis by clearing protein aggregates and damaged organelles, degrading collagen levels, and preventing excessive matrix protein accumulation in the kidney, thereby alleviating inflammation and delaying the progression of interstitial fibrosis [44, 45]. Research by Jun Li et al. has confirmed that kidney damage and renal fibrosis can be treated by relieving oxidative stress damage, regulating autophagy, and other mechanisms [46]. In this study, after treatment with dioscin, the expression levels of fibrosis-related proteins TGF-β, FN, and Collagen I were significantly reduced. However, after knocking down NRF2, the regulatory effect of dioscin on these proteins was inhibited. This indicates that dioscin may treat renal fibrosis by alleviating oxidative stress and promoting protective autophagy, and this therapeutic effect is NRF2-dependent.

Numerous studies have demonstrated a complex interaction between oxidative stress and autophagy. In recent years, extensive research has confirmed that ROS generated during oxidative stress can induce autophagy [47]. After activation, autophagy in the body can reduce the level of ROS by degrading damaged organelles and proteins within cells, thereby regulating the balance between oxidation and antioxidation [48]. However, in most pathological conditions, autophagy tends to reduce oxidative stress levels [49]. Research has confirmed that traditional Chinese medicine can treat UAN and RF by enhancing cellular and tissue autophagy [50, 51]. However, in this experiment, we observed that compared with normal cells, the UA group cells showed a lower level of autophagy. This contrasts with the experiment conducted by Yan Hu et al. [52]. According to Yan Hu et al.‘s research, it has been shown that higher levels of UA induce an increased level of autophagy in the HK-2 cells model. This suggests that the high concentration of uric acid may trigger compensatory autophagy. By comparing the experiments and modeling conditions, we consider that the difference lies in the concentration of UA and the duration of its action in the modeling process. We used a concentration of 0.5 mg/mL of UA for modeling for 24 h, which has a shorter time and lower concentration compared to other people’s experimental methods [38]. By comparison, this may have reduced the level of protective autophagy in our cell model and did not reach the conditions for excessive autophagy. However, it should be noted that we were unable to ascertain whether dioscin can effectively modulate autophagy in vivo to regulate HUA, which will be a future research objective.

In conclusion, our data demonstrate that dioscin can effectively improve the structural and functional changes of UA induced-HK-2 cells fibrosis model. Mechanistically, dioscin activates the P62-KEAP1-NRF2 antioxidant pathway to prevent excessive ROS and activates protective autophagy to treat fibrosis. In addition, dioscin exerts its effects by regulating the P62-KEAP1-NRF2 signaling pathway in an NRF2-dependent manner. These results suggest that dioscin may be a promising therapeutic option for renal fibrosis.

## Electronic supplementary material

Below is the link to the electronic supplementary material.

Supplementary Material 1

## Data Availability

All relevant data are within the paper.

## Abbreviations

HUA: Hyperuricemia
UA: Uric acid
UAN: Uric acid nephropathy
RF: Renal fibrosis
ROS: Reactive oxygen species
MDC: Monodansylcadaverine
