# Supplementary material for: Dioscin regulates oxidative stress and autophagy in uric acid-induced HK-2 cells through the P62-KEAP1-NRF2 signaling pathway
Source: BMC Nephrol. 2025 Jul 29;26:422. doi: 10.1186/s12882-025-04311-z (PMC12309051; doi:10.1186/s12882-025-04311-z)
Supplement: Supplementary file 1 — Supplementary Material 1 [file 12882_2025_4311_MOESM1_ESM.pptx]

## Slide 1
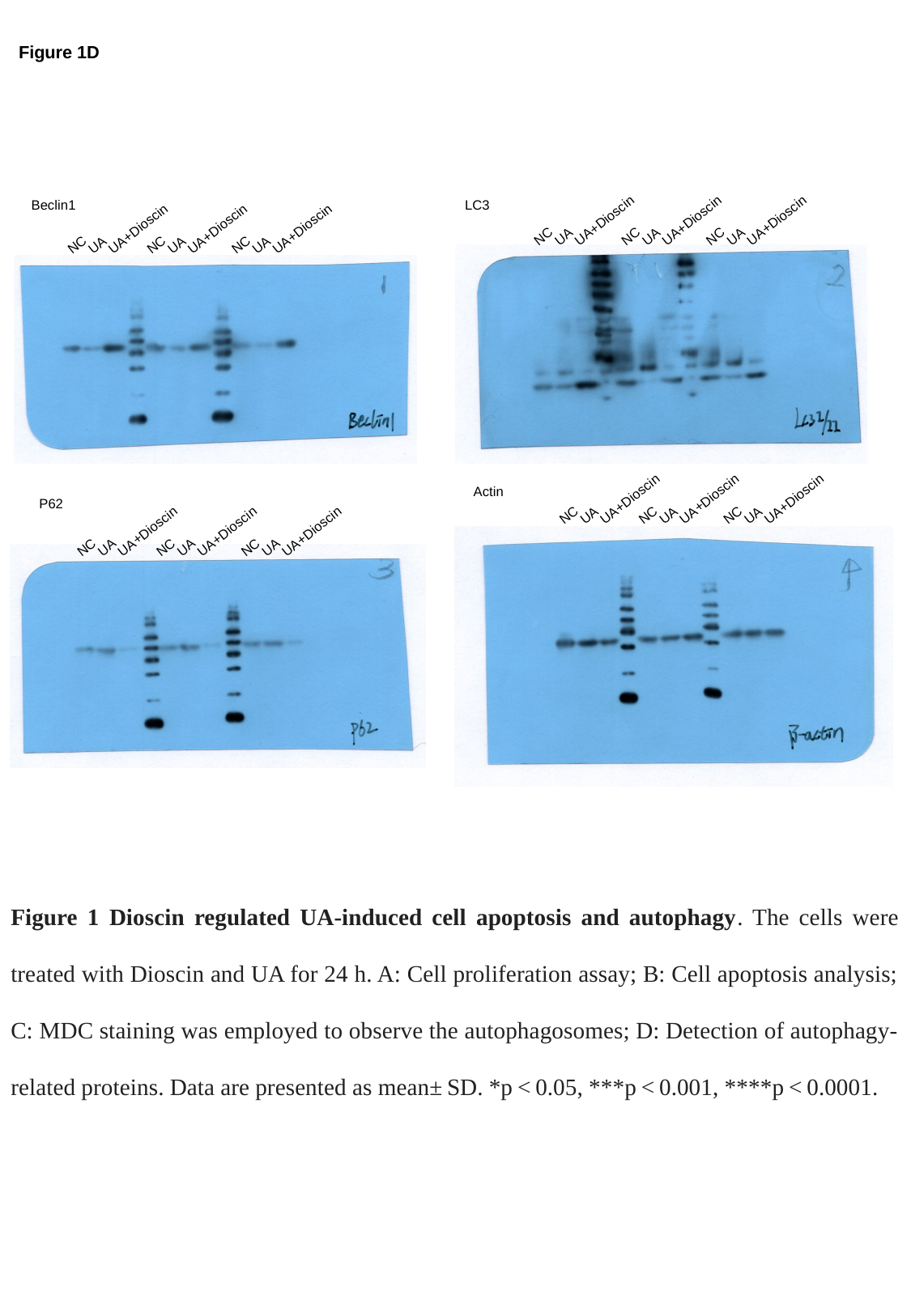

Figure 1D
Beclin1
LC3
UA+Dioscin
UA+Dioscin
UA+Dioscin
UA
UA
UA
NC
NC
NC
UA+Dioscin
UA+Dioscin
UA+Dioscin
UA
UA
UA
NC
NC
NC
Actin
UA+Dioscin
UA+Dioscin
UA+Dioscin
UA
UA
UA
NC
NC
NC
P62
UA+Dioscin
UA+Dioscin
UA+Dioscin
UA
UA
UA
NC
NC
NC
Figure 1 Dioscin regulated UA-induced cell apoptosis and autophagy. The cells were treated with Dioscin and UA for 24 h. A: Cell proliferation assay; B: Cell apoptosis analysis; C: MDC staining was employed to observe the autophagosomes; D: Detection of autophagy-related proteins. Data are presented as mean± SD. *p < 0.05, ***p < 0.001, ****p < 0.0001.

## Slide 2
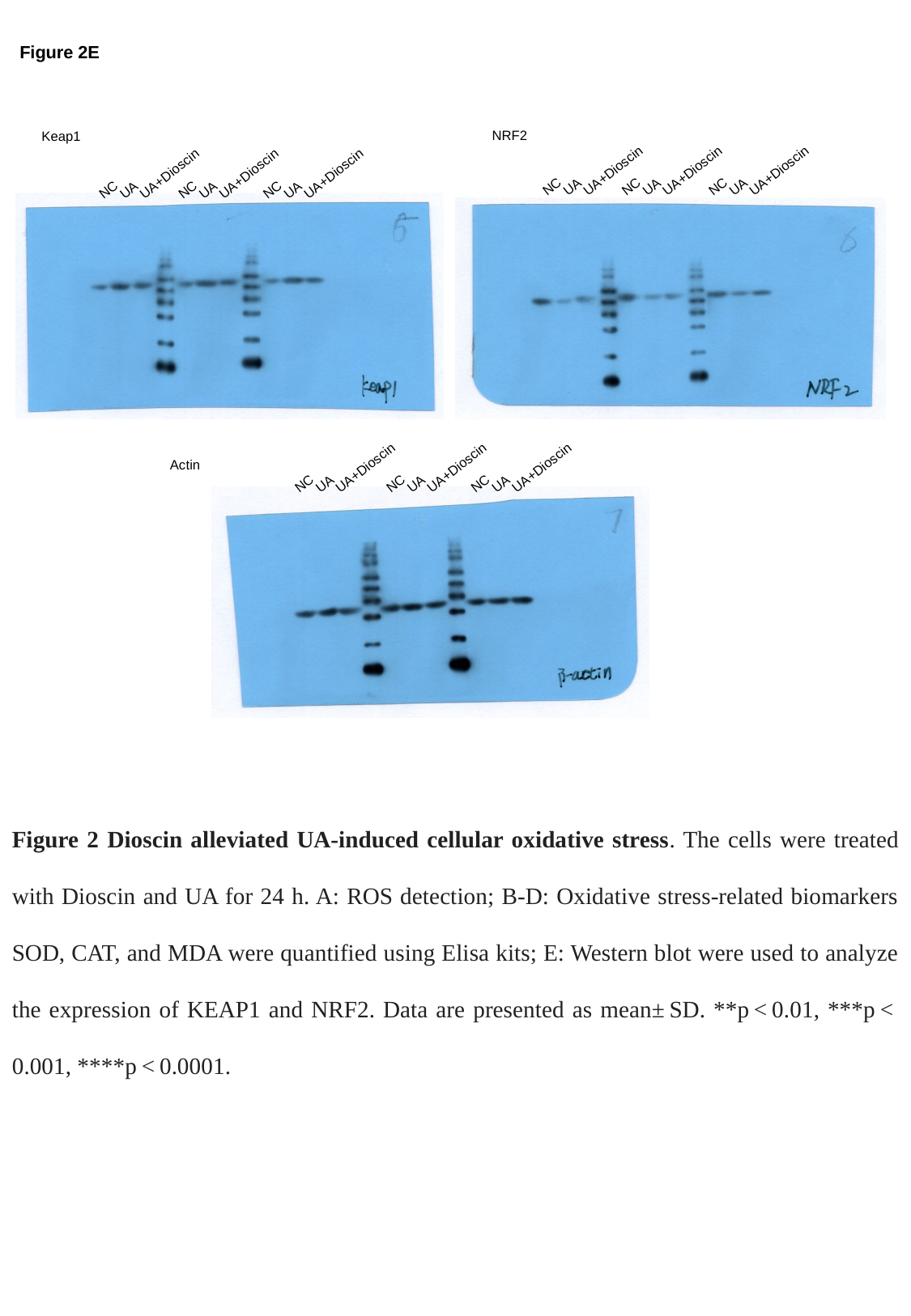

Figure 2E
NRF2
Keap1
UA+Dioscin
UA+Dioscin
UA+Dioscin
UA
UA
UA
UA+Dioscin
UA+Dioscin
UA+Dioscin
UA
UA
UA
NC
NC
NC
NC
NC
NC
UA+Dioscin
UA+Dioscin
UA+Dioscin
Actin
UA
UA
UA
NC
NC
NC
Figure 2 Dioscin alleviated UA-induced cellular oxidative stress. The cells were treated with Dioscin and UA for 24 h. A: ROS detection; B-D: Oxidative stress-related biomarkers SOD, CAT, and MDA were quantified using Elisa kits; E: Western blot were used to analyze the expression of KEAP1 and NRF2. Data are presented as mean± SD. **p < 0.01, ***p < 0.001, ****p < 0.0001.

## Slide 3
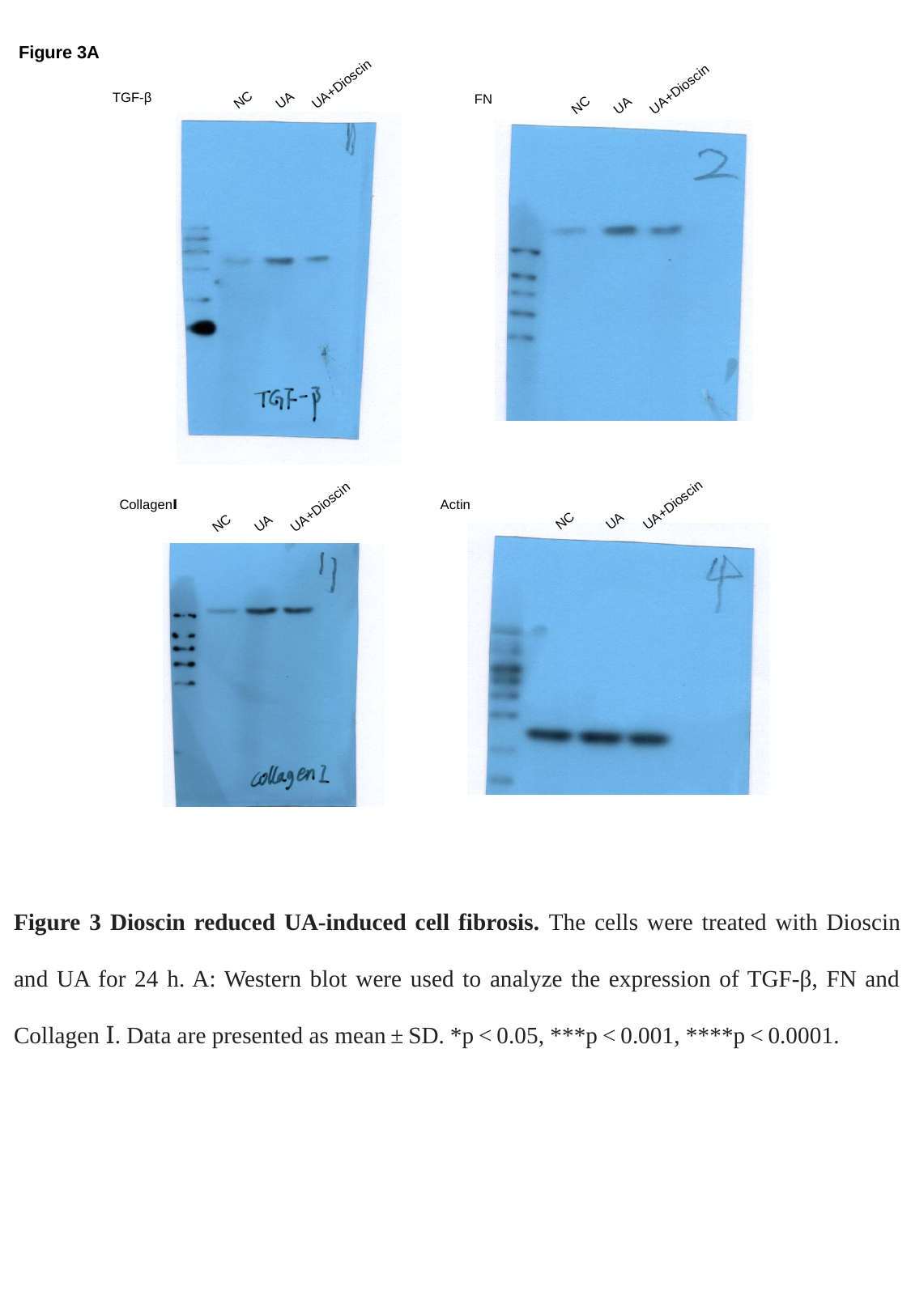

Figure 3A
UA+Dioscin
UA
UA+Dioscin
NC
UA
NC
TGF-β
FN
UA+Dioscin
UA
UA+Dioscin
CollagenⅠ
Actin
UA
NC
NC
Figure 3 Dioscin reduced UA-induced cell fibrosis. The cells were treated with Dioscin and UA for 24 h. A: Western blot were used to analyze the expression of TGF-β, FN and Collagen Ⅰ. Data are presented as mean ± SD. *p < 0.05, ***p < 0.001, ****p < 0.0001.

## Slide 4
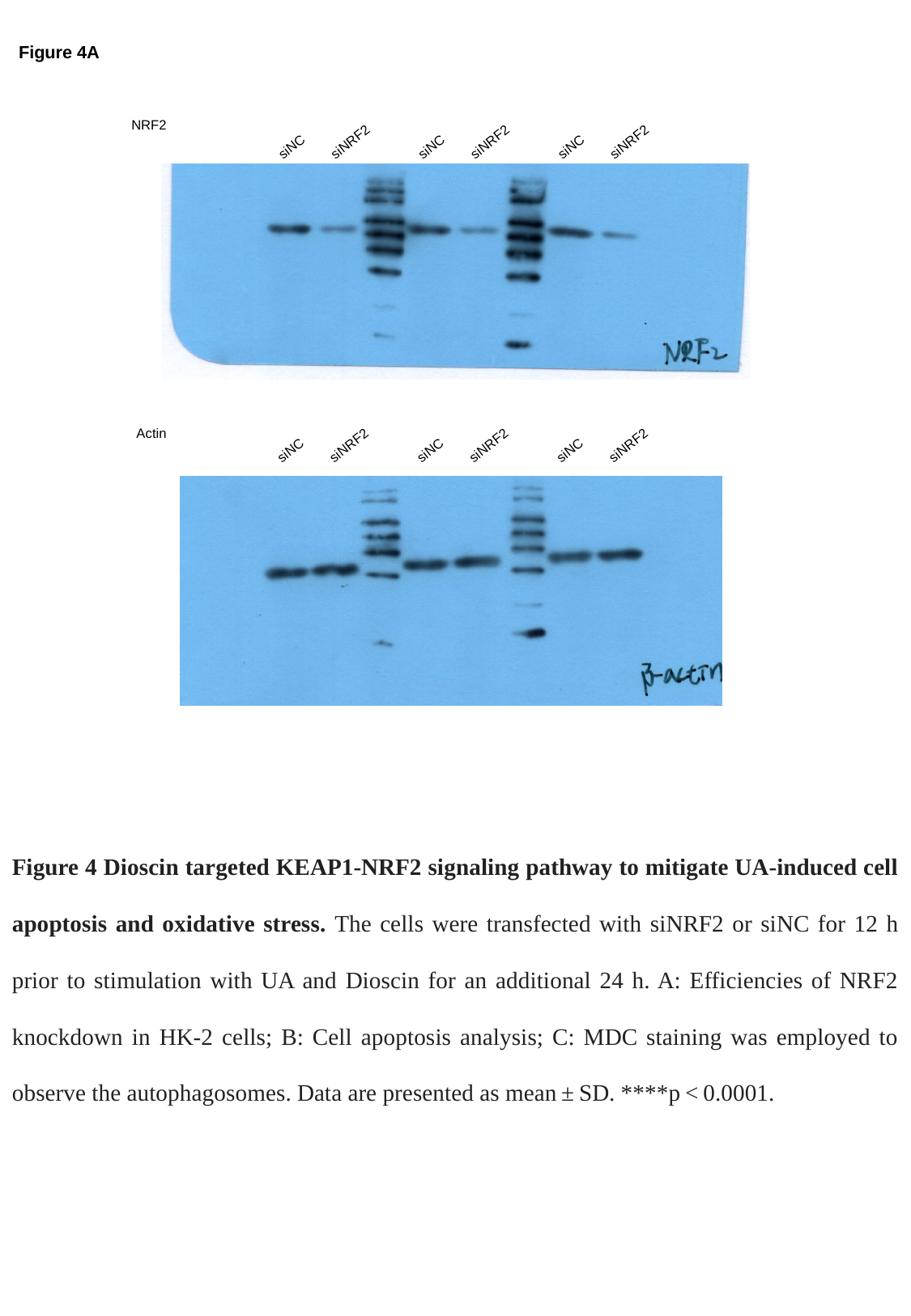

Figure 4A
NRF2
siNC
siNRF2
siNC
siNRF2
siNC
siNRF2
Actin
siNC
siNRF2
siNC
siNRF2
siNC
siNRF2
Figure 4 Dioscin targeted KEAP1-NRF2 signaling pathway to mitigate UA-induced cell apoptosis and oxidative stress. The cells were transfected with siNRF2 or siNC for 12 h prior to stimulation with UA and Dioscin for an additional 24 h. A: Efficiencies of NRF2 knockdown in HK-2 cells; B: Cell apoptosis analysis; C: MDC staining was employed to observe the autophagosomes. Data are presented as mean ± SD. ****p < 0.0001.

## Slide 5
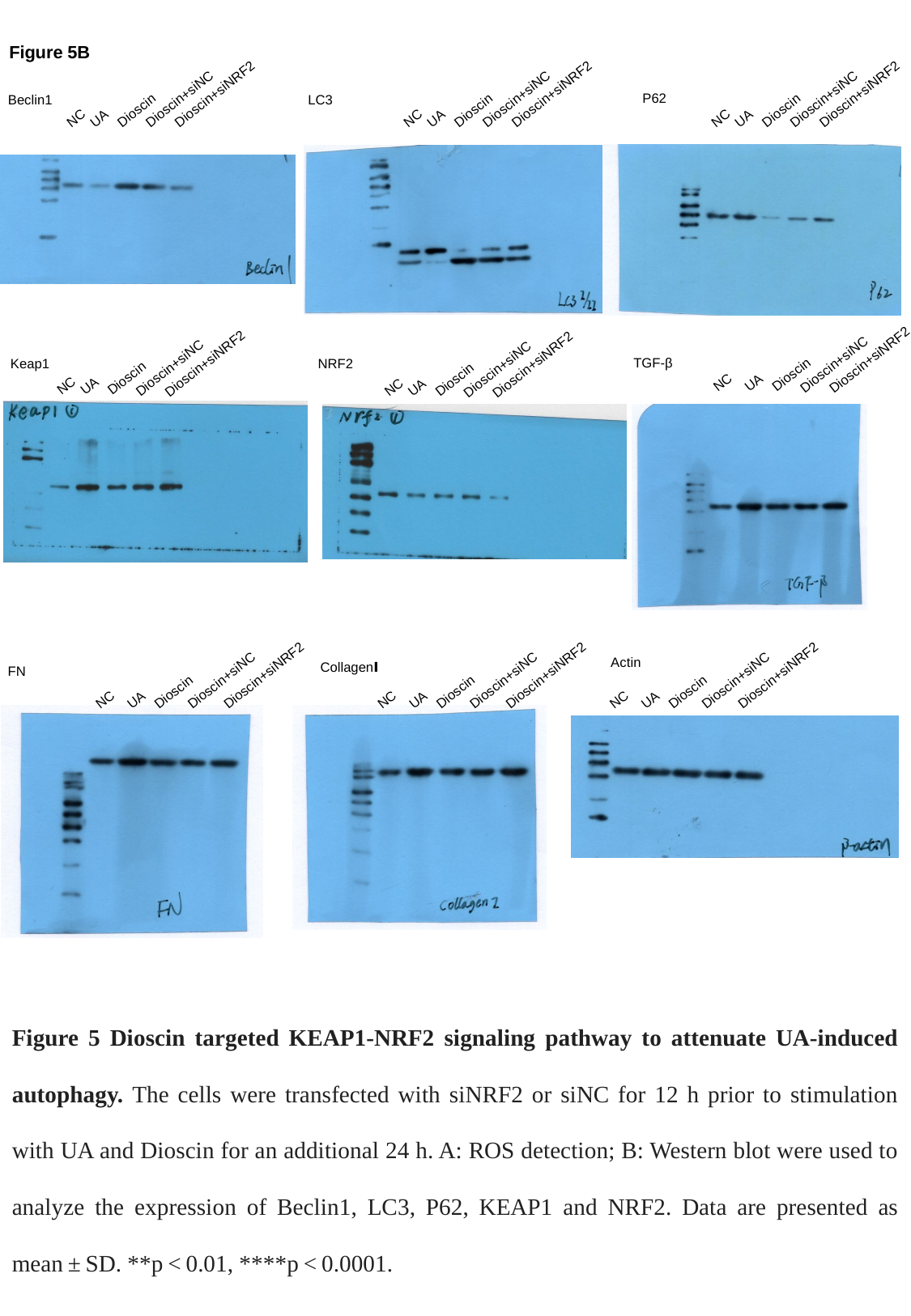

Figure 5B
Dioscin+siNRF2
Dioscin+siNRF2
Dioscin+siNRF2
P62
Dioscin
Dioscin+siNC
Dioscin
Dioscin+siNC
Dioscin
Dioscin+siNC
UA
UA
UA
Beclin1
LC3
NC
NC
NC
Dioscin+siNRF2
Dioscin+siNRF2
Dioscin
TGF-β
Dioscin+siNRF2
NRF2
UA
Dioscin+siNC
Keap1
Dioscin
UA
Dioscin+siNC
Dioscin
NC
UA
Dioscin+siNC
NC
NC
Actin
CollagenⅠ
FN
Dioscin+siNRF2
Dioscin+siNRF2
Dioscin+siNRF2
Dioscin
Dioscin+siNC
Dioscin
Dioscin+siNC
Dioscin
Dioscin+siNC
UA
UA
UA
NC
NC
NC
Figure 5 Dioscin targeted KEAP1-NRF2 signaling pathway to attenuate UA-induced autophagy. The cells were transfected with siNRF2 or siNC for 12 h prior to stimulation with UA and Dioscin for an additional 24 h. A: ROS detection; B: Western blot were used to analyze the expression of Beclin1, LC3, P62, KEAP1 and NRF2. Data are presented as mean ± SD. **p < 0.01, ****p < 0.0001.
